# Transcriptomic Analysis of *Taar5* Expression and Co-Expression Networks in the Cerebellum During Perinatal Development

**DOI:** 10.3390/brainsci15080791

**Published:** 2025-07-25

**Authors:** Anastasia N. Vaganova, Ramilya Z. Murtazina, Anna B. Volnova, Vassiliy Tsytsarev, Alena B. Karaseva, Evgeniya V. Efimova, Raul R. Gainetdinov

**Affiliations:** 1 Institute of Translational Biomedicine, St. Petersburg State University, Universitetskaya nab. 7/9, St. Petersburg 199034, Russia; 2 Department of Neurobiology, University of Maryland School of Medicine, 20 Penn St., HSF-2, Baltimore, MD 21201, USA; 3 Federal State Budgetary Scientific Institution «Institute of Experimental Medicine» (FSBSI «IEM»), Str. Academica Pavlova 12, St. Petersburg 197022, Russia

**Keywords:** TAAR5, trace amine-associated receptors, cerebellum, development, dopamine, transcriptomic data

## Abstract

**Background:** Dopamine participates in the cognitive cerebellar role and in cerebellum development. The trace amine-associated receptor (TAARs, TAAR1-TAAR9) system contributes to dopamine signaling tuning. So, the aim of the present study is the analysis of the TAARs’ gene expression and functional associations in prenatal and neonatal mouse cerebellums. **Methods:** The transcriptomic data represented in the GEO repository was performed to identify Taars expression and co-expression patterns in embrionic and postnatal mouse cerebellum. **Results:** Open transcriptomic data analysis showed cerebellar expression of the *Taar5* gene mRNA both in prenatal and early postnatal samples. The identified *Taar5* expression was confirmed by RT-PCR in P5 mice. We identified the association between *Taar5* expression and the expression of proliferation-related genes in late prenatal E13.5 samples, which was replaced by co-expression with genes involved in metabolism in P5–6 samples. These associations are suggested to mirror the previously identified *Taar5* expression in Purkinje cells, which proliferate at the E13.5 and mature in the postnatal period. However, the analysis of TAAR5 co-expression with markers of different cell populations revealed the pronounced co-expression of TAAR5 in the P5–6 cerebellum with microglial markers, which is shifted to the association with astroglial markers in P10. **Conclusions:** The *Taar5* gene was found to be active in the cerebellum samples taken around birth, and its co-expression pattern differs in the embryo stage and the early days after birth. We suggest that the Taar5 receptor may be involved in cerebellum development; however, further research is necessary to elucidate its role in this process.

## 1. Introduction

G-protein-coupled trace-amine-associated receptors (TAARs), at least TAAR1, TAAR2, and TAAR5, are known modulators of dopaminergic neurotransmission [1,2,3,4]. Previous studies have identified TAAR1 expression in the cerebellum [5], and the analysis of public human transcriptomic data also suggests TAAR5 expression in the cerebellum [6]. Previously, TAAR5 mRNA expression in the adult human cerebellum and early postnatal mice cerebellum samples were identified in publicly available transcriptomic datasets [7]. In mice, TAAR5 expression was revealed in the Purkinje cells and the medial vestibular nucleus, and TAAR5 gene knockout in mice lowered endurance but improved coordination and balance compared to wild-type controls [7]. Also, the rat cerebellar granule cell layer binds 3-iodothyronamine [6], which is the ligand both for TAAR1 [8,9] and TAAR5 [10,11,12,13].

Considering the involvement of the TAAR5 receptor in the modulation of the dopamine (DA) signaling system [1,14] and Purkinje cells’ functioning [6], and the possible participation of the TAAR5 receptor in DA system development and regulation [1], the scope of the present study is the assessment of mouse *Taar5* and the expression of other *Taars* in the developing cerebellum and the study of their function by in silico analysis.

The cerebellum is a relatively small but neuron-rich part of the brain, containing [15,16] nearly 80% of the brain’s neurons [14,17]. In addition to its involvement in motor and cognitive functions, the cerebellum, in close association with the basal ganglia, modulates reward circuitry, emotion, and social behavior via the pathways to the striatum, prefrontal cortex, and thalamus, and via monosynaptic inputs to the ventral tegmental area DA neurons [18,19,20]. The vermis is suggested to be part of the limbic circuit [21], and has been associated with basic emotions such as fear, supported by connections with the amygdala. The posterior cerebellar hemispheres are associated with complex emotions and social interactions and are connected with the prefrontal cortex [14,18].

Cerebellar development starts in humans early, during the first trimester of pregnancy (30 days post-conception) and lasts until the 2 years postnatal age [18,22]. In rodents, the cerebellum matures completely at postnatal day P21 [23,24,25,26]. Additionally, about 20% of infants born before 32 weeks exhibit cerebellar injury and volume loss, with the incidence increasing in those born at less than 28 weeks. Cerebellar underdevelopment is associated with cerebral palsy, epilepsy, intellectual disabilities, and autism spectrum disorders [22,27].

The output of the cerebellar cortex occurs via functionally heterogeneous inhibitory Purkinje cells [28]. These cells emerge between E10.5 and E13.5 [29], with consequent partial loss of population by apoptosis between E15 and P10 [23]. NMDA receptor blocking and GABAA receptor activation by ethanol [24] or propofol [25] in the early postnatal period resulted in a reduction in the Purkinje cell number and their dendrite length in line with the disruption of Bergmann glia development and consequent delay of granule neuron migration to the cerebellar cortex internal granular layer [25,26].

There is evidence of functional interactions between TAAR1 and the dopaminergic system [3,4,30,31]. The studies of TAAR5 gene knockout mice also demonstrate changes in the DA signaling system [13]. In the rat cerebellum, DA immunoreactivity presents a uniform distribution pattern [15], and all the dopaminergic receptor subtypes, DRD1-DRD5, are expressed in the cerebellum [15,32]. Several studies demonstrated the involvement of the cerebellum in DA-related neurological and psychiatric disorders, such as schizophrenia, autistic spectrum disorders, and drug addiction [15,18,33], as well as Parkinson’s disease [15]. Extrinsic dopaminergic fibers, which innervate the cerebellum, originate from the midbrain dopaminergic cell groups of the ventral tegmental area and, to a lesser extent, from the retrorubral nucleus and substantia nigra [31].

The output of the cerebellum occurs via functionally heterogeneous inhibitory Purkinje cells [28]. These cells emerge between E10.5 and E13.5 [29] with consequent partial loss of population by apoptosis between E15 and P10 [23]. DA production in Purkinje also varies during ontogeny. The TH expression appears in the Purkinje neurons at P8 in the cerebellar vermis. It increases at P13–P15 with a consequent reduction at P19 and then increases again after 1 month of age, reaching a maximum expression at 11 months [34].

There are several pieces of evidence for DA involvement in cognitive cerebellar functions. In particular, changes in DRD2 expression levels in mouse Purkinje cells alter sociability and preference for social novelty without affecting motor functions [32]. In humans, reduced cerebellar DRD2 expression is related to the negative symptoms of schizophrenia and autistic spectrum disorders [15]. Simultaneously, DRD1 expression in the lateral nucleus is involved in navigation memory, response inhibition, working memory, and prepulse inhibition (PPI) [19].

No previous studies have examined the role of trace-amine-associated receptors in embryogenesis or postnatal ontogenesis. Taking into consideration the possible significance of DA signaling in the cerebellum’s development, especially in the early postnatal period, we studied the TAARs’ expression and functions in this structure at different developmental stages.

## 2. Materials and Methods

### 2.1. Public Transcriptomic Data

The expression profiles of cerebellar tissue were received from publicly available transcriptome datasets. RNA sequencing data were searched in the National Center of Biotechnology Information (NCBI) Gene Expression Omnibus (GEO) [35]. RNAseq-generated datasets that met the following criteria were selected: (1) complete TAAR expression data in raw counts, reads per kilobase million (RPKM), fragments per kilobase million (FPKM), or transcripts per million (TPM); (2) four or more biological replicates per study group; (3) mouse samples; and (4) because of low TAAR mRNA transcription levels, only datasets consisting of samples with the minimum number of reads in SRA files > 35 million spots. After excluding irrelevant datasets, five datasets generated by RNAseq for the entire cerebellum were included in the review (Table 1).

### 2.2. Data Normalization and Statistical Analysis

As TPM normalization is considered to be suitable for comparing multiple samples from different experiments [36] and for the correlation analysis [37], raw RNAseq counts and FPKM/RPKM-normalized data were downloaded and converted to TPM.

Raw counts were converted to TPM by the following formula:

$$TPM=\frac{q_{i}/l_{i}}{\Sigma_{j}\left(q_{j}/l_{j}\right)}\times 10^{6}$$

where qi denotes reads mapped to the transcript, li is the transcript length, and $\Sigma_{j}\left(q_{j}/l_{j}\right)$ is the sum of mapped reads to the transcript normalized by transcript length.

Additional TPM normalization was performed for RPKM/FPKM data, as it was previously identified that TPM outperforms RPKM in co-expression analysis [38]. The following formula [38] was applied for the conversion:

$$TPM=\frac{FPKM}{\Sigma FPKM}\times 10^{6}$$

TPM values above the threshold level of 0.1 were considered positive (following [39] and [40]). The distribution of TPM-normalized expression levels in the analyzed samples was visualized using the ggplot2 R package [41].

### 2.3. Gene Co-Expression Measurement and Pathway Enrichment Analysis

Data for different study groups were analyzed independently. *Taar5* co-expressed genes were selected by Spearman’s correlation coefficient (ρ > 0.3, *p* < 0.05). The comparative analysis of the selected clusters was performed as described below.

GO enrichment analysis (identification of Gene Ontology (GO) terms [42] that were significantly enriched by the genes of the selected set) was performed in the identified co-expressed gene clusters. GO biological process terms were applied for this analysis. The Kyoto Encyclopedia of Genes and Genomes (KEGG) pathway enrichment analysis also was performed. Enrichment analysis and visualization of results were performed by the clusterProfiler (version 4.10.1) Bioconductor package [43], which is designed to perform over-representation analysis (ORA), i.e., to determine which a priori defined gene sets are more present [43]. The enrichGO and enrichKEGG functions with default arguments were applied; all genes listed in the database were used as the background. GO term enrichment is prone to bias due to the hierarchical relationships between GO terms which are ignored in ORA and leads to redundant terms in the results [43,44]. Simultaneously, the KEGG database is curated better than GO but is likely to be incomplete [44]. Thus, we used both approaches to overcome these limitations.

Additionally, we applied ORA for brain cell signatures that were received from the BRETIGEA (version 1.0.3) R package [45] and adopted them for the murine transcriptome. Cell marker enrichment for the identified clusters was performed by the clusterProfiler (version 4.10.1) R package, applying the enricher function for universal enrichment [43].

### 2.4. Taar5 mRNA Expression Analysis by RT-PCR

C57BL/6 mice (P5) were obtained from the Saint Petersburg State University vivarium. Both male and female C57BL/6N mice (P5) were used (*n* = 5 and 4, respectively). Mice were euthanized by decapitation. Tissues were dissected on ice, immediately frozen in liquid nitrogen, and stored at −80 °C. RNA isolation from the whole cerebellum was performed using TRI Reagent (MRC, Houston, TX, USA) according to the manufacturer’s instructions. The RNA pellet was resuspended in RNase-free water and kept at −80 °C until used. RNA concentration was quantified using spectrophotometry (NanoDrop, München, Germany), and 1 μg of RNA was taken for the synthesis of cDNA using Revertaid Reverse Transcriptase (Thermo Scientific, Waltham, MA, USA) with a total reaction volume of 30 μL. To eliminate any remaining genomic DNA, the TURBO DNA-free kit (Thermo Scientific, USA) was used. As a control for the successful removal of genomic DNA, each sample was exposed to the same treatment, except that the reverse transcriptase was not added (NRT control). A total of 1 μL of cDNA was used for PCR. The reaction product was amplified using qPCRmix-HS SYBR (Evrogen, Moscow, Russia) by qPCR (CFX96, Bio-Rad, Hercules, CA, USA) with primers for *mouse Taar1*, *Taar2*, *Taar5*, *Taar6*, and *Taar9* genes [46]. Considering the high expression stability in the mouse cerebellum and its suitability as an internal marker of mRNA integrity and the normalization of mRNA expression [47,48], the mouse *Gapdh* was used as a housekeeping reference gene as described previously [7]. RNA isolated from the main olfactory epithelium, which is known to express *Taar5* [46,49], was used as a positive control. The CFX Manager software (version 3.1) was applied for Ct determination and melt curve analysis. The amplification specificity was confirmed by melting curve analysis (from 55 to 95 °C) and 2% agarose gel electrophoresis in sodium borate buffer. Normalization and analysis were performed using the ΔΔCt method.

## 3. Results

### 3.1. Taar5 mRNA Expression in Perinatal Mouse Cerebellum Samples in RNA-Seq-Generated GEO Data

**Figure 1:**
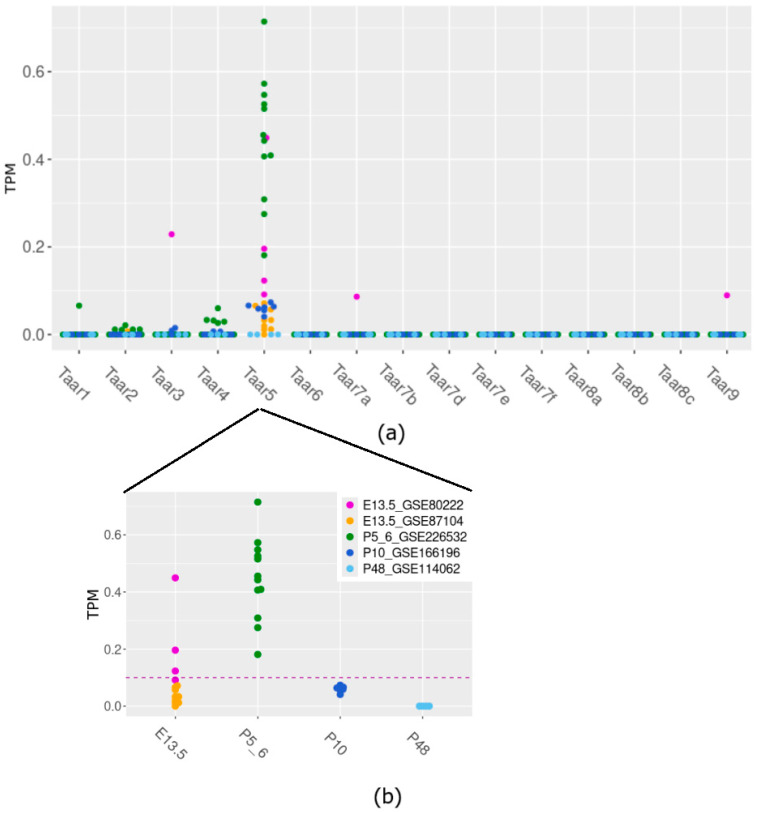
TAARs’ mRNA expression in murine cerebellar samples acquired at different development time points (**a**) and detailed *Taar5* expression at different developmental stages (**b**).

The analysis of transcriptomic RNA-generated datasets shows that cerebellar samples of E13.5, P5–6, and P10 mice consistently express only the *Taar5* gene, among other TAARs. In specimens collected on E13.5 (25%) and P5–6 (100%), *Taar5* expression exceeds the cutoff value of 0.1 TPM (Figure 1a).

On the other hand, *Taar5* (Figure 1b) is reproducibly expressed in the P10 cerebellum at the sub-cutoff values, unlike other *Taar* genes mRNA, which were not identified in most studied samples in the same dataset. In most studied samples in the same dataset, other *Taar* genes’ mRNA was not identified. To prevent batch bias, we refrained from statistically estimating the differences between developmental stages, since the data for different datasets were obtained by distinct laboratories under dissimilar conditions.

**Figure 2:**
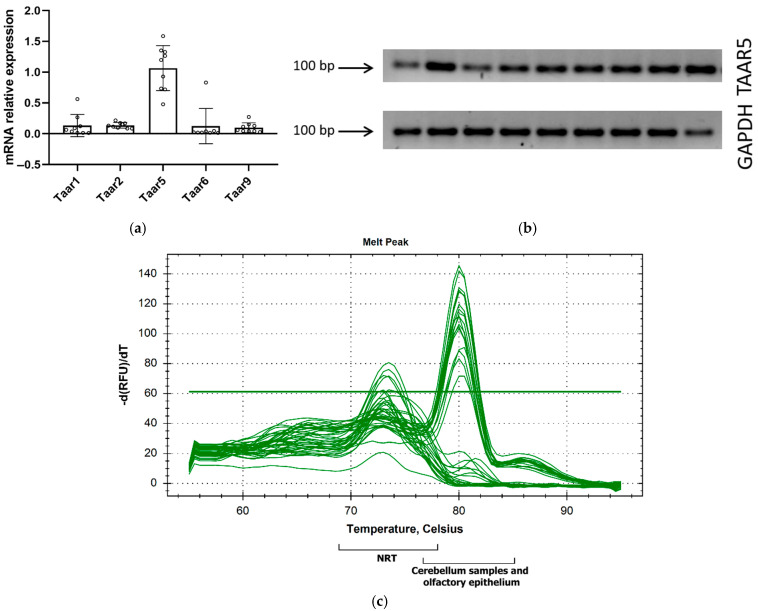
Reverse transcription–polymerase chain reaction (RT-qPCR) confirmed primary *Taar5* mRNA expression in the cerebellums of P5 mice (**a**); *Taar5* mRNA RT–PCR amplification products were separated on a 2% agarose gel and resulted in a single product with the desired length (**b**) and a single melt peak in a qPCR melt curve analysis at 80 °C (**c**). NRT—no reverse transcriptase control.

We further assessed *Taar* genes’ expression in the cerebellums of P5 mice using RT-qPCR (Figure 2a).

Since GSE226532 represented *Taar5* expression in cerebellar samples harvested both from male and female P5 mice [50], we also included male (*n* = 5) and female (*n* = 4) samples in the in vivo study. According to published RNAseq data, *Taar5* gene mRNA is prominently expressed in the cerebellum in the early postnatal period compared to other *Taars*, and our data supported the *Taar5* mRNA presence in all studied samples (Figure 2b,c).

### 3.2. Taar5 Functional Associations in Prenatal Cerebellum Samples

**Figure 3:**
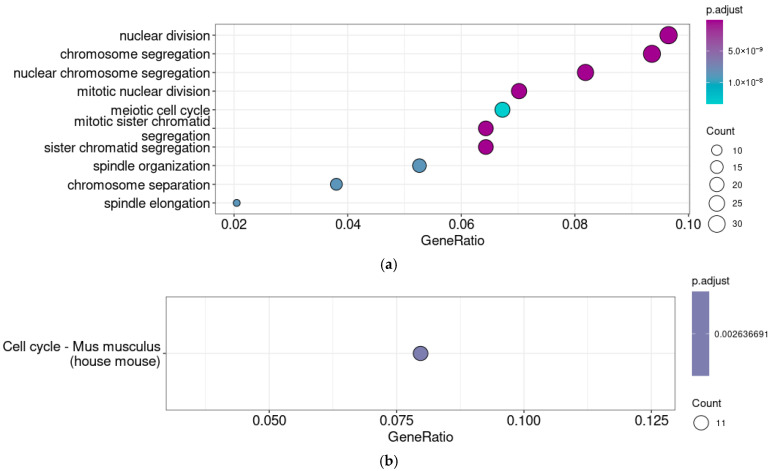
Functional analysis of genes co-expressed with *Taar5* in normally developing mice cerebellar samples at E13.5. GO biological process ontology terms (**a**) and KEGG pathway (**b**) enrichment analysis (GSE87104).

To clarify the functional associations of *Taar5* expression in mouse prenatal cerebellar samples on the E13.5, we analyzed 364 (ρ > 0.3, *p* < 0.05) *Taar5*-associated genes using the GO term enrichment test (i.e., identifying the functional groups of genes that were over-represented in the gene set) and the KEGG pathway enrichment test (Figure 3a,b).

The results of two enrichment tests in *Taar5*-associated gene clusters identified in E13.5 (GSE87104) specimens were congruent and pointed out the associations between *Taar5* gene expression and proliferation. *Taar5* shows the co-expression of genes involved in the “Cell cycle” KEGG pathway and genes associated with GO terms such as “Nuclear division,” “Chromosome segregation,” “Spindle organization,” etc.

These results are determined first of all by genes whose products are involved in chromosome segregation (i.e., *Incenp*, *Knstrn*, *Kif23*, *Syce1l*, *Prc1*, *Mei4*, *Kif18b*, *Espl1*, *Spc24*, *Ncaph*, *Spag5*, *Bub1b*, *Chek2*, *Ncapd2*). However there are also transcriptional factor genes *Msx2* and *Nkx3-1*, or genes involved in cell proliferation regulation like *Birc5*, *Anxa1*, and *Epgn*.

Additionally, we performed the enrichment analysis to identify the overrepresentation of BRETIGEA-derived cell markers in *Taar5* co-expressed gene clusters. However, no statistically significant results were received, possibly because the embryonic data were not included in the BRETIGEA design [45].

### 3.3. Taar5 Functional Associations in Postnatal P5–6 Cerebellum Samples

**Figure 4:**
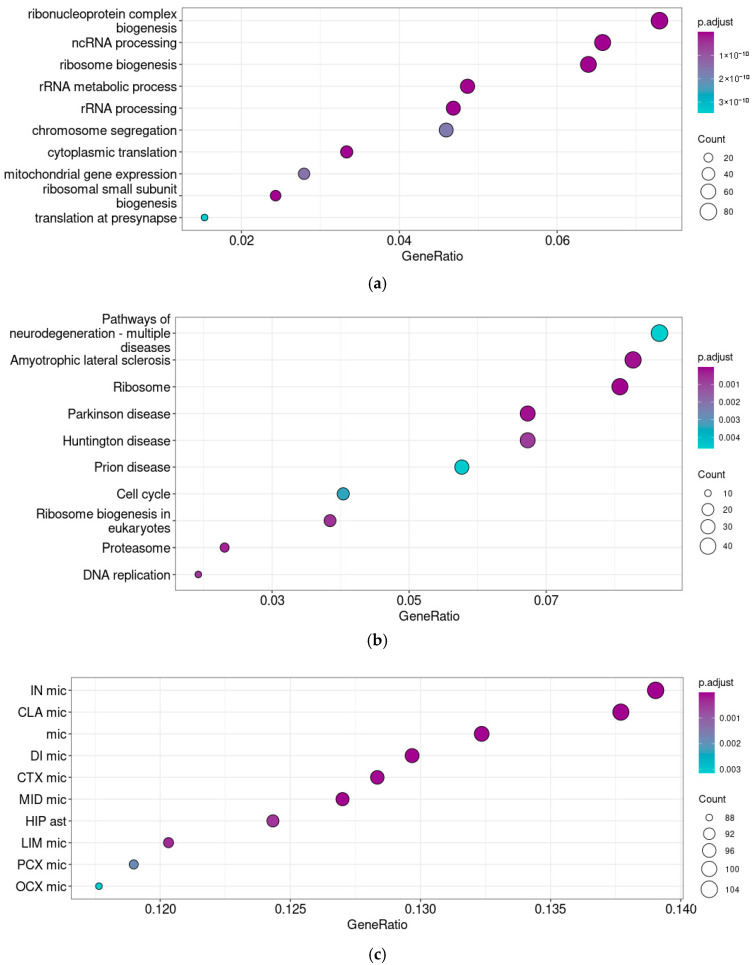
Functional analysis of genes co-expressed with *Taar5* in normally developing mice cerebellar samples at P5–6. GO biological process ontology terms (**a**), KEGG pathway (**b**), and BRETIGEA neuronal cells’ mRNA signatures enrichment analyses (**c**). The cell types are named following the BRETIGEA R package [45] nomenclature: CLA—claustrum, CTX—cortex, DI—diencephalon, HIP—hippocampus, IN—insula, LIM—limbic cortex, MID—midbrain, OCX—occipital cortex, PCX—parietal cortex, ast—astrocytes, mic—microglia.

The correlation analysis identified 1156 genes co-expressed with *Taar5* in P5–6 cerebellar samples. A switch of the *Taar5* co-expressed gene set accompanies the maturation of the cerebellum on postnatal days P5–6 (GSE226532). Both enrichment tests identified the co-expression of *Taar5* mRNA with genes involved in ribosome biogenesis (Figure 4a), including 19 genes of proteins of the small ribosome subunit, 27 genes of large ribosome subunit components, and 19 nuclear *Mrp* nuclear genes of the mitochondrial ribosomes. At the same time, 47 genes involved in proteasome development and function were identified in the same gene cluster.

KEGG pathway enrichment analysis also identified associations with several neurodegenerative diseases, like amyotrophic lateral sclerosis, Huntington’s disease, or prion diseases, in the selected gene cluster (Figure 4b). The detailed analysis of enrichment cores revealed that this association caused *Taar5* co-expression with mitochondrial genes, which was also confirmed by the GO terms’ enrichment patterns, tubulins, and genes involved in the response to oxidative stress. Supplementary S1 represents the mapping of relevant enrichment cores on KEGG pathways.

Genes co-expressed with *Taar5* in the P5–6 samples were enriched by microglial markers. In this cluster (Figure 4c), we found an association of *Taar5* mRNA expression with astroglial markers as well.

### 3.4. Taar5 Functional Associations in Postnatal P10 Cerebellum Samples

**Figure 5:**
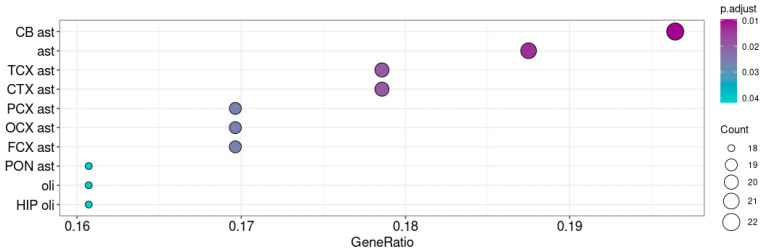
Enrichment of neuronal cells’ mRNA signatures in genes co-expressed with *Taar5* in normally developing cerebellums at P10 (GSE133196). The cell types are named following BRETIGEA R package (McKenzie et al., 2018 [45]) nomenclature: CB—cerebellum, CTX—cortex, FCX—frontal cortex, OCX—occipital cortex, PCX—parietal cortex, PON—pons, ast—astrocytes, oli—oligodendrocytes.

Despite the low levels of *Taar5* expression in P10 samples (GSE166196), we also performed the enrichment analysis of 250 genes (ρ > 0.3, *p* < 0.05) co-expressed with *Taar5* in this developmental stage. After adjusting the *p* value, we did not identify any significantly enriched gene sets in these clusters, but we still found some weak associations with genes involved in catabolism and development. However, in P10 samples, the *Taar5* gene was co-expressed with astroglial markers, especially with markers of cerebellar astroglia (Figure 5).

### 3.5. The Taar5 Co-Expressed Gene Pattern Gradually Changes from the Embryonal to Postnatal Stages

**Figure 6:**
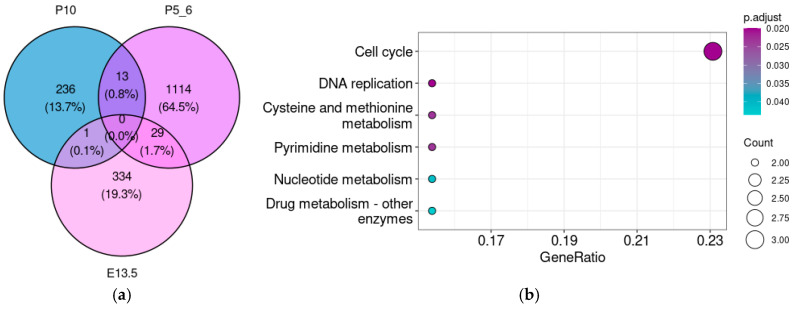

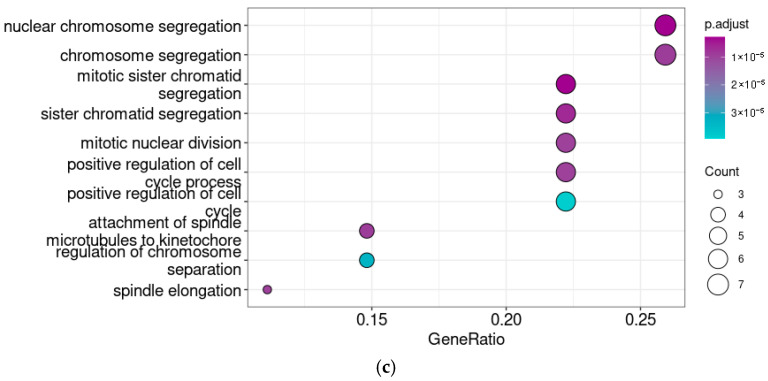
Venn diagram (**a**) demonstrates the overlap between *Taar5* co-expressed genes at different developmental stages. Functional analysis of genes co-expressed with *Taar5* in the cerebellar samples of normally developing mice both at E13.5 and P5–6. KEGG pathway (**b**) and GO biological process ontology term (**c**) enrichment analysis.

For a more comprehensive assessment of associations that might be overlooked when analyzing each stage individually, we estimated the overlap between genes co-expressed in different development stages (Figure 6a). Although *Taar5* co-expression patterns differed significantly across developmental stages, several common genes appeared in both E13.5 and P5–6 and in the P5–6 and P10 samples.

The genes involved in cell cycle and division are enriched in the gene set that is co-expressed with *Taar5* both on E13.5 and P5–6 (Figure 6b,c). However, no significant enrichment results were identified in the 29 genes that were reproducibly co-expressed with *Taar5* both in P5–6 and P10.

## 4. Discussion

Trace-amine-associated receptors’ functions in the neural tissue are studied fragmentarily. Previously, the involvement of the *Taar5* gene product in the natural progenitor cell proliferation was suggested [1,51]. In our study, we reveal the expression of *Taar5* mRNA both in late embryonic and early postnatal mouse cerebellum samples. The identified expression pattern of *Taar5* is collinear with previously reported data [7]. However, we did not observe the stable and reproducible expression of any other *Taar* genes in the mouse cerebellum, possibly because of the insufficient sequencing depth, as previously discussed for the *Taar6* gene, whose expression in the mouse brain remains uncertain [52].

In the late embryonic period at E13.5, we identified the co-expression of the *Taar5* gene with genes involved in cell cycle and division. This stage is accompanied by the proliferation of Purkinje cells [53]. Granule cell precursors, highly proliferative neurons, are also present in the cerebellum in the late embryonic stage [54]. Therefore, it is impossible to concretize if *Taar5* is involved in some specific cell group’s proliferation in late embryogenesis or the regulation of the cell cycle in several groups of neuronal or glial progenitors. Also, no association with particular cells’ molecular signature was identified at this stage.

Later cerebellum development stages are associated with switches in the functional characteristics of *Taar5* co-expressed genes. At P5–6, Purkinje cells finish forming a plate, and dendritic arborization initiates in these cells [55]. Instead, granule cell precursors undergo massive proliferation [29]. The identified associations can be interpreted under the previously identified expression of *Taar5* in Purkinje cells, which mature or undergo apoptosis rather than proliferate. The *Taar5* co-expressed genes at this stage are involved both in proliferation, like at the E13.5 stage, and in the biosynthetic and energetic metabolism-associated processes, especially with mitochondrial functioning. Cerebellum development requires normal mitochondrial functioning and is prone to mitochondrial diseases, which are commonly associated with impaired cerebellum morphology and movement [56,57]. In particular, the clinical data indicated major involvement of the cerebellum in mitochondrial disease with movement disorders. This might be related to the particular vulnerability of the cerebellum to energy deficiency [55].

Additionally, we reveal the association of *Taar5* co-expressed genes with the response to reactive oxygen species and oxidative stress. The identified correlations between *Taar5* and energy metabolism [58] may also explain this association. Also, the transitory production of reactive oxygen species in the developing cerebellar cortex is required for normal cerebellar development. Previous research revealed that inhibiting reactive oxygen species leads to morphologic changes in the cerebellum and alters motor behavior [59]. All these trends strongly suggest that *Taar5* may be associated with some morphogenetic processes in the cerebellum, which need energy and complex biomolecules.

GO term enrichment analysis identified another functional aspect of the *Taar5* gene’s co-expressed cluster for central nervous system cell gene signatures. Simultaneously, in the normally developing cerebellum, *Taar5* is co-expressed with microglial markers and, to a lesser extent, with astroglial markers. This relationship does not designate the *Taar5* expression in any group of glial cells, but this needs to be evaluated in further studies. Cerebellar microglial cells also proliferate in the first three weeks of postnatal development [60] and are involved both in synapse development and in triggering apoptosis in immature Purkinje cells in the postnatal cerebellum [60,61]. A specific astrocyte group called Bergmann glia also participates in postnatal cerebellum development [25,62].

On the day P10, granule neurons migrate from the external granular layer to the internal granular layer during P8 to P10 [25], massive Purkinje cell death slows down [23], and some cells, like microglia, still proliferate [61]. The GO terms or KEGG pathway enrichment tests did not provide significant results in the *Taar5* co-expressed gene cluster at this stage. Genes involved in energetic metabolism regulation (including genes of the Insulin Resistance KEGG pathway) and morphogenesis showed trends of overrepresentation.

It has now been shown that dopaminergic transmission is closely linked to the work of trace amine receptors [3,4,15]. In adult mice, the TAAR5 receptor is expressed primarily in DA-producing Purkinje cells [7]. These cells also express DRD1, DRD2, DRD3, and DRD5 DA receptors [31,62]. The role of the cerebellar DA system remains largely unexplored. Meanwhile, tyrosine hydroxylase (TH) immunoreactive Purkinje neurons modulate cerebellar cognitive functions like spatial navigation memory or working memory [31]. All the DA receptor subtypes (DRD1-DRD5) were identified in the cerebellar neurons [16,31]. Simultaneously, genetic knockout of D1 receptors in Bergmann glia, which play an important role in the development of the cerebellum, leads to decreased locomotor activity and impaired social activity [63].

To sum up, *Taar5* is expressed in the prenatal and neonatal cerebellum. Most likely, it is significant for the Purkinje cell’s development and maturation, but its involvement in glia development and functioning also is quite probable. Using a transcriptomic-based approach to study cerebellar *Taar5* expression dysregulation may shed light on their role in the pathogenesis of DA-related mental diseases, including schizophrenia. Previously, the association of *TAAR5* gene polymorphisms with cognitive deficit in patients with schizophrenia was described in the literature [64]. At the same time, animal studies demonstrated that TAAR5 agonist α-NETA causes significant alterations of the gamma rhythm of brain activity [65] and sensory gating [66] in a manner consistent with schizophrenia-related deficits. Further study of the role played by TAAR5 in the cerebellum will undoubtedly contribute to progress in understanding the mechanisms of mental diseases. Thus, TAAR5 may be a promising therapeutic target for neuropsychiatric disorders.

The findings of this study have to be seen in light of some limitations. We have limited options for comparing data obtained independently in different research groups. Only a few datasets with the limited study groups in the GEO [67] repositories were relevant to this study. However, it was impractical to include datasets with lower sequencing depths because TAARs seem to have low expression. This study included transcriptomic datasets obtained in various laboratories, and standardizing them was not possible, so we applied TPM normalization to compare different datasets. Despite this, the identified TAAR5 expression levels were low, so the identified associations may be prone to bias and should be interpreted with caution, especially for P10 mice. This approach provides us with a result, which we could confirm in vivo, at least for genes that have known human orthologs and are valuable in terms of translation studies, i.e., TAAR1, 2, 5, 6, and 9. Thus, only a semi-quantitative assessment is feasible, as conducting a comparative statistical analysis to identify age-dependent expression differences could yield unreliable results. Additionally, we decided to abandon the confirmation of our results by the estimation of TAAR5 expression on the protein levels due to low TAAR5 mRNA expression, which suggests there is also a low protein level in combination with the lack of a highly specific antibody. As described previously, the GPCR antibodies frequently have low specificity [68].

The chosen method provided the information for co-expressed genes but could not clarify the role of TAAR5 in the identified processes. However, assigning gene function based on its co-expressed partners is considered a valuable and emerging method [69]. The identified associations and their functional significance need further study and experimental validation.

Previously, in adult animals, the influence of TAAR5 on motor function and its expression in the cerebellum have been revealed. Our study serves as a pilot investigation aimed at understanding whether the TAAR system is active in the cerebellum during earlier developmental stages. Our findings provide a foundation for further exploration of the role of TAARs in neurodevelopment. This study underscores the importance of considering the TAAR system in future investigations of cerebellar development and function, particularly in the context of monoaminergic regulation.

## 5. Conclusions

Only the *Taar5* gene, but not the other genes of the trace-amine-associated receptor family, is stably and pronouncedly expressed in the embryonic and early postnatal samples. Previously, *Taar5* expression was demonstrated in human and mouse cerebellums. However, the data for the receptor expression are indirect because this evidence was received in TAAR5 knockout mice by LacZ labeling [7]. In this study, we aimed to describe this gene expression, alongside other trace-amine-associated receptor genes, in developing mouse cerebellums in the later embryonic development and postnatal days P2–P10. Then, we analyzed the functional significance of genes that are co-expressed with *Taar5* in embryonic or juvenile mouse cerebellums. We observed that, as cerebellum development progresses, the functional associations between *Taar5* and other genes change. The identified functional shifts may be interpreted following previously identified *Taar5* expression in Purkinje cells, which are proliferating in the embryonic cerebellum when *Taar5* is co-expressed with genes involved in proliferation and maturation in the early postnatal period when *Taar5* is co-expressed with genes of the catabolic and anabolic pathways. At the same time, *Taar5* expression in the studied cerebellum samples is associated with the expression of glial cell signatures. Further studies on separated cell fractions could elucidate whether the TAAR5 receptor is expressed in cerebellar DA-producing cells or other cell populations.

Ongoing research of the effect of pharmacological modulation of TAAR5 activity or conditional TAAR5 knockout on cerebellum development will allow for this receptor’s potential as a prospective therapeutic target for the correction of cerebellum development disabilities, for example, disorders related to prenatal ethanol exposure, to be revealed.

## Figures and Tables

**Table 1:** Characteristics of RNA-seq-generated datasets included in the review.

GEO ID	Title	Mouse Strain	Age	n	Available Data
GSE80222	Polycomb Ezh2 controls the fate of GABAergic neurons in the embryonic cerebellum	C57BL/6	E13.5	4 *	RPKM
GSE87104	RNA-seq Analysis of Wild Type and Ptpn11-deficient cerebellar Transcriptomes	CD1	E13.5	9 *	Raw counts
GSE226532	Ethanol Induced Cerebellar Transcriptomic Changes in a Postnatal Model of Fetal Alcohol Spectrum Disorders: Focus on Disease Onset	C57BL/6	P5–6	12 *	Raw counts
GSE166196	Divergent and overlapping hippocampal and cerebellar transcriptome responses following developmental ethanol exposure during the secondary neurogenic period.	C57BL/6	P10	7 *	Raw counts
GSE114062	Comparison between a mouse line selected for high voluntary wheel running and control line in two brain regions, cerebellum and striatum	Hsd:ICR	P48	4	FPKM

* the number of control, wild-type, and non-treated samples included in the analysis.

## Data Availability

The data are available in the GEO database (https://www.ncbi.nlm.nih.gov/geo/ (accessed on 5 October 2024), the detailed information is listed in Table 1).

## Supplementary Materials

The following supporting information can be downloaded at: https://www.mdpi.com/article/10.3390/brainsci15080791/s1, Supplementary S1: KEGG pathways for neurodegenerative diseases with mapped genes co-expressed with the *Taar5* gene in mouse cerebellar samples obtained on days P5–6 of postnatal development. Supplementary S2: Full-length agarose gel: reverse transcription–polymerase chain reaction with the TAAR5 and GAPDH (housekeeping gene) mRNA-specific primers using RNA isolated from the cerebellums of P5 mice.
