# Supplementary material for: Transcriptomic Analysis of Taar5 Expression and Co-Expression Networks in the Cerebellum During Perinatal Development
Source: Brain Sci. 2025 Jul 25;15(8):791. doi: 10.3390/brainsci15080791 (PMC12384425; doi:10.3390/brainsci15080791)

SUPPLEMENTARY S1

KEGG pathways for neurodegenerative diseases with mapped genes co-expressed with *Taar5* gene in mouse cerebellar samples obtained on P5-6 days of postnatal development.

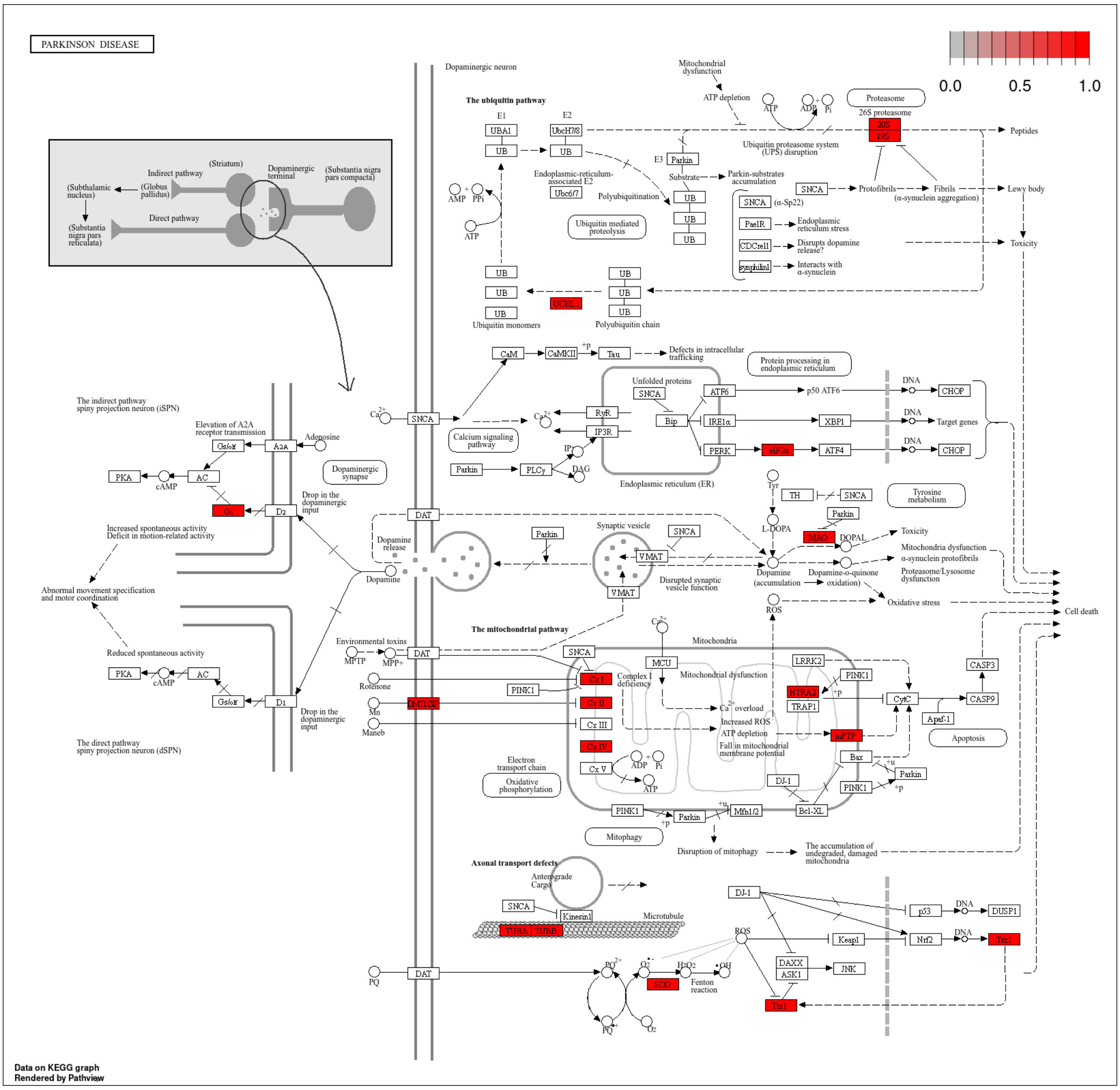

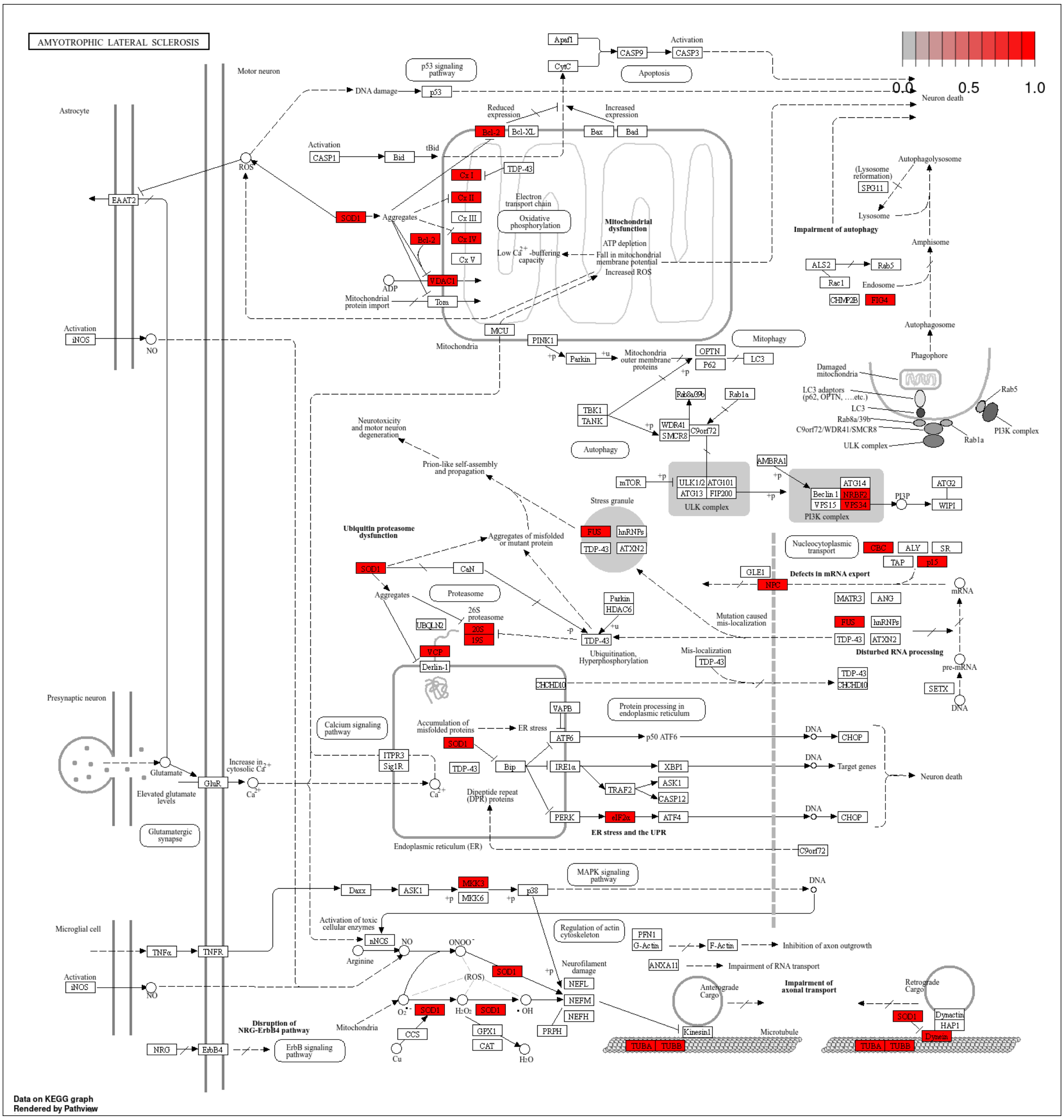

AMYOTROPHIC LATERAL SCLEROSIS
0.0
0.5
1.0
Astrocyte
Motor neuron
EAAT2
ROS
DNA damage
p53
p53 signaling pathway
Apaf1
CytC
CASP9
CASP3
Activation
Apoptosis
Neuron death
Reduced expression
Increased expression
Bcl-2
Bcl-XL
Bax
Bad
Activation
CASP1
Bid
tBid
Cx I
TDP-43
Cx II
Electron transport chain
Cx III
Oxidative phosphorylation
SOD1
Aggregates
Bcl-2
Cx IV
Cx V
Mitochondrial dysfunction
ATP depletion
Fall in mitochondrial membrane potential
Increased ROS
Low Ca2+-buffering capacity
ADP
VDAC1
Mitochondrial protein import
Tom
MCU
Mitochondria
Activation
iNOS
NO
PINK1
Parkin
Mitochondria outer membrane proteins
OPTN
P62
LC3
Mitophagy
TBK1
TANK
Rab8a/39b
Rab1a
WDR41
SMCR8
C9orf72
Autophagy
mTOR
ULK1/2
ATG101
ATG13
FIP200
ULK complex
AMBRA1
ATG14
Beclin 1
NRBF2
VPS15
VPS34
PI3K complex
PI3P
ATG2
WIPI
Neurotoxicity and motor neuron degeneration
Prion-like self-assembly and propagation
Stress granule
FUS
hnRNPs
TDP-43
ATXN2
Ubiquitin proteasome dysfunction
Aggregates of misfolded or mutant protein
SOD1
CaN
Aggregates
Proteasome
26S proteasome
20S
19S
UBQLN2
VCP
Derlin-1
Parkin
HDAC6
TDP-43
Ubiquitination, Hyperphosphorylation
Mutation caused mis-localization
Mis-localization
TDP-43
CHCHD10
Nucleocytoplasmic transport
GLE1
NPC
CBC
ALY
SR
TAP
p15
Defects in mRNA export
mRNA
MATR3
ANG
FUS
hnRNPs
TDP-43
ATXN2
Disturbed RNA processing
pre-mRNA
SETX
DNA
(Lysosome reformation)
SPG11
Autophagolysosome
Lysosome
Impairment of autophagy
Amphisome
ALS2
Rab5
Rac1
Endosome
CHMP2B
FIG4
Autophagosome
Phagophore
Damaged mitochondria
LC3 adaptors (p62, OPTN, ...etc.)
LC3
Rab8a/39b
C9orf72/WDR41/SMCR8
ULK complex
Rab5
Rab1a
PI3K complex
Presynaptic neuron
Glutamate
Elevated glutamate levels
GluR
Increase in cytosolic Ca2+
Ca2+
Glutamatergic synapse
Calcium signaling pathway
ITPR3
Sig1R
TDP-43
Accumulation of misfolded proteins
ER stress
SOD1
Bip
VAPB
Protein processing in endoplasmic reticulum
ATF6
p50 ATF6
IRE1α
XBP1
TRAF2
ASK1
CASP12
PERK
eIF2α
ATF4
Dipeptide repeat (DPR) proteins
ER stress and the UPR
Endoplasmic reticulum (ER)
C9orf72
DNA
CHOP
Target genes
Neuron death
Daxx
ASK1
MKK3
MKK6
p38
MAPK signaling pathway
Microglial cell
TNFα
TNFR
Activation of toxic cellular enzymes
nNOS
NO
Arginine
ONOO-
(ROS)
SOD1
Neurofilament damage
NEFL
NEFM
NEFH
PRPH
Regulation of actin cytoskeleton
PFN1
G-Actin
F-Actin
Inhibition of axon outgrowth
ANXA11
Impairment of RNA transport
Activation
iNOS
NO
O2
SOD1
H2O2
SOD1
• OH
CCS
Cu
GPX1
CAT
H2O
Mitochondria
Disruption of NRG-ErbB4 pathway
NRG
ErbB4
ErbB signaling pathway
Anterograde Cargo
Kinesin1
TUBA
TUBB
Microtubule
Impairment of axonal transport
Retrograde Cargo
SOD1
Dynactin
HAP1
Dynein
TUBA
TUBB
Data on KEGG graph
Rendered by Pathview

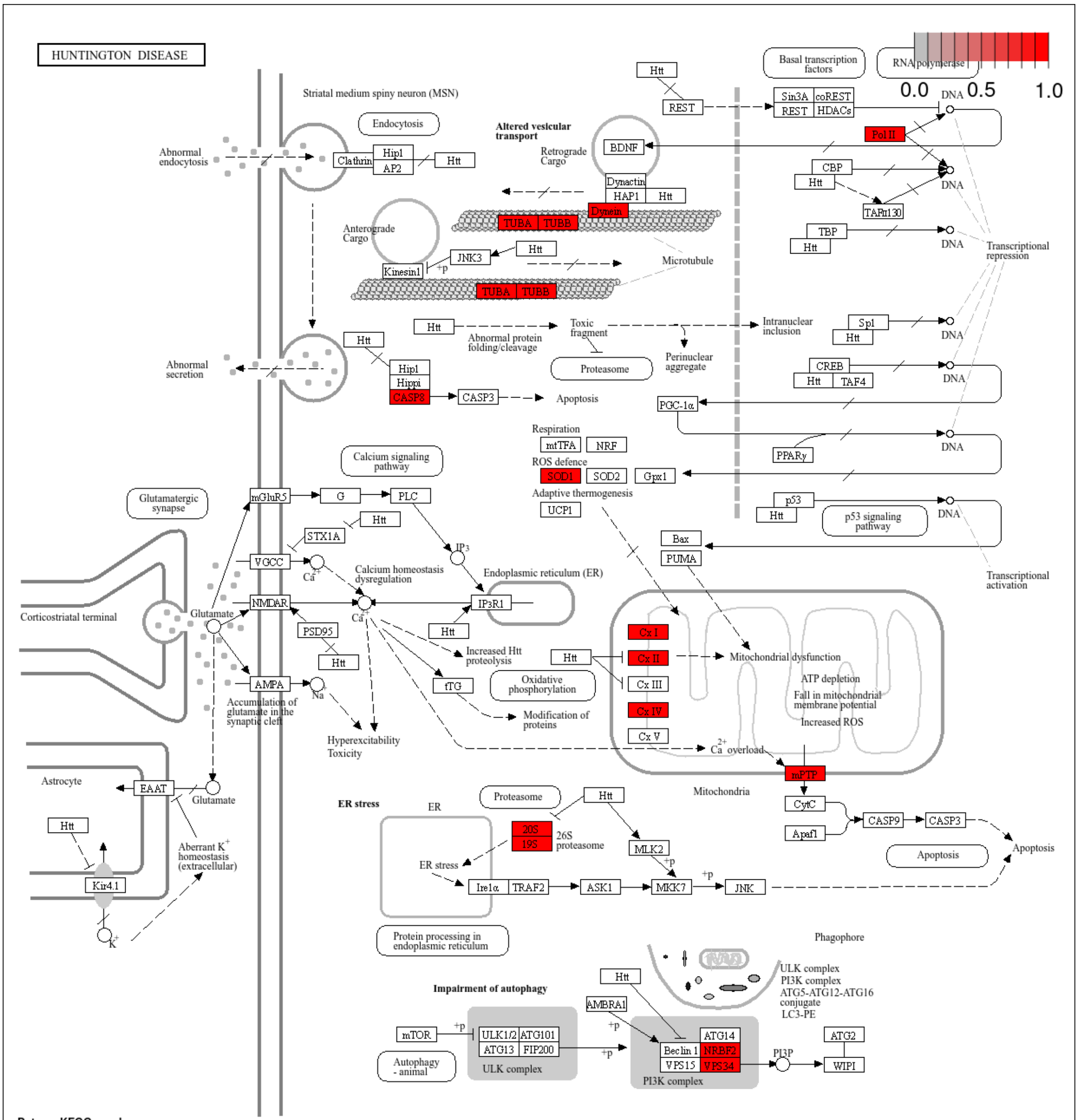

Data on KEGG graph
Rendered by Pathview

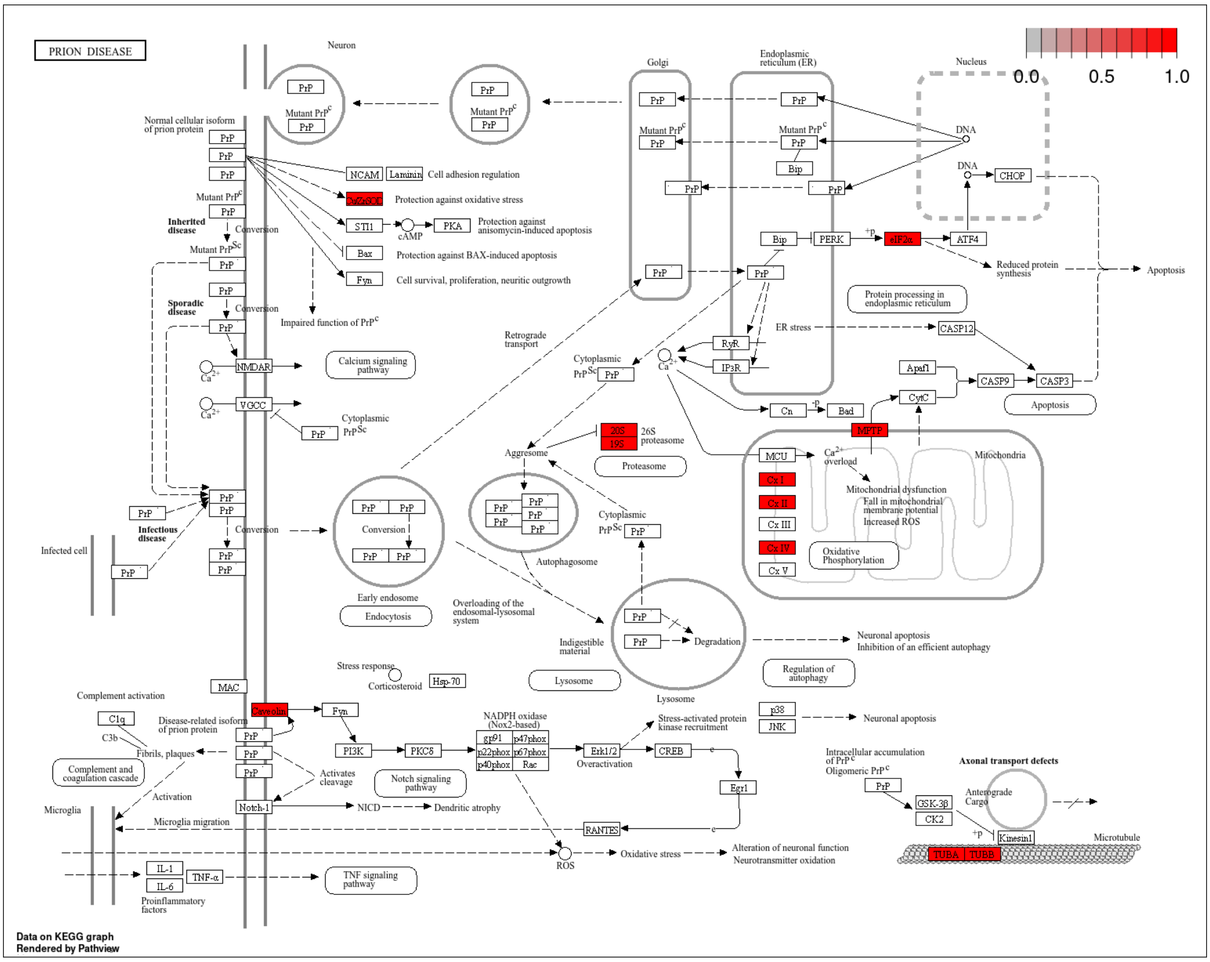
PRION DISEASE
Neuron
Golgi
Endoplasmic reticulum (ER)
Nucleus
0.0
0.5
1.0
PrP
Mutant PrP
Normal cellular isoform of prion protein
NCAM
Laminin
Cell adhesion regulation
Protection against oxidative stress
STI1
cAMP
PKA
Protection against anisomycin-induced apoptosis
Bax
Protection against BAX-induced apoptosis
Fyn
Cell survival, proliferation, neuritic outgrowth
Inherited disease
Conversion
Sporadic disease
Impaired function of PrP
DNA
Bip
CHOP
PERK
ATF4
Reduced protein synthesis
Apoptosis
Protein processing in endoplasmic reticulum
ER stress
CASP12
RyR
IP3R
Retrograde transport
Cytoplasmic PrP
NMDAR
Calcium signaling pathway
VGCC
Apaf1
CASP9
CASP3
CytC
Cn
Bad
MPTP
20S
19S
26S proteasome
Proteasome
Aggresome
MCU
Ca2+ overload
Mitochondria
Cx I
Cx II
Cx III
Cx IV
Cx V
Mitochondrial dysfunction
Fall in mitochondrial membrane potential
Increased ROS
Oxidative Phosphorylation
Infectious disease
Infected cell
Early endosome
Endocytosis
Autophagosome
Overloading of the endosomal-lysosomal system
Indigestible material
Degradation
Lysosome
Neuronal apoptosis
Inhibition of an efficient autophagy
Regulation of autophagy
Stress response
Corticosteroid
Hsp-70
MAC
Complement activation
C1q
C3b
Disease-related isoform of prion protein
Fibrils, plaques
Complement and coagulation cascade
Activation
Microglia
Microglia migration
PI3K
PKCδ
NADPH oxidase (Nox2-based)
gp91
p47phox
p22phox
p67phox
p40phox
Rac
Erk1/2
Overactivation
CREB
Stress-activated protein kinase recruitment
p38
JNK
Egr1
RANTES
Activates cleavage
Notch signaling pathway
Notch-1
NICD
Dendritic atrophy
ROS
Oxidative stress
Alteration of neuronal function
Neurotransmitter oxidation
Intracellular accumulation of PrP
Oligomeric PrP
Axonal transport defects
GSK-3β
CK2
Anterograde Cargo
Kinesin1
Microtubule
TUBA
TUBB
IL-1
IL-6
TNF-α
TNF signaling pathway
Proinflammatory factors
Data on KEGG graph
Rendered by Pathview

SUPPLEMENTARY S2

Full-length agarose gel: reverse transcription-polymerase chain reaction with *TAAR5* and *GAPDH* (housekeeping gene) mRNA specific primers using RNA isolated from the cerebellum of P5 mice. 1-9, mouse cerebellum; K+, mouse olfactory epithelium; K-, negative control (water). Red is a source of cropped image.

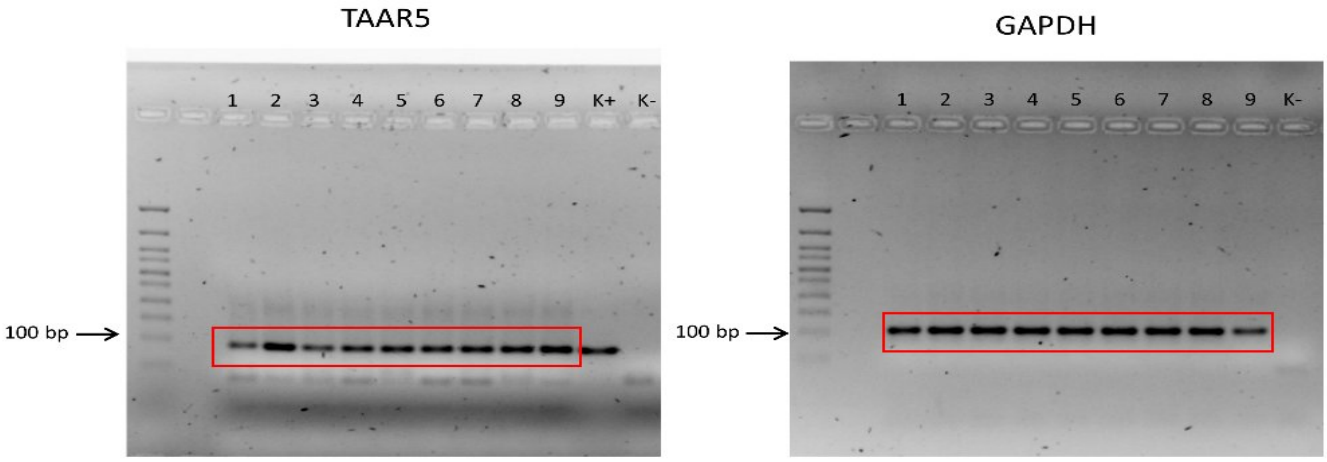

Supplement: Supplementary file 1 [file brainsci-15-00791-s001.zip › brainsci-3713792-supplementary.pdf]
